# Cefaclor-induced hypersensitivity: Differences in the incidence of anaphylaxis relative to other 2^nd^ and 3^rd^ generation cephalosporins

**DOI:** 10.1371/journal.pone.0254898

**Published:** 2021-07-22

**Authors:** Hyo-In Rhyou, Young-Hee Nam, Su-Chin Kim, Go-Eun Doo, Chae-Yeon Ha, Hee-Joo Nam, Sung-Dae Woo, Youngsoo Lee, Jae-Hyuk Jang, Hyun-Young Lee, Young-Min Ye

**Affiliations:** 1 Department of Internal Medicine, College of Medicine, Dong-A University, Busan, Korea; 2 Dong-A Regional Pharmacovigilance Center, Dong-A University Hospital, Busan, Korea; 3 Clinical Trial Center, Ajou University Hospital, Suwon, Korea; 4 Ajou Regional Pharmacovigilance Center, Ajou University Hospital, Suwon, Korea; 5 Department of Allergy and Clinical Immunology, Ajou University School of Medicine, Suwon, Korea; Xiamen University - Malaysia Campus: Xiamen University - Malaysia, MALAYSIA

## Abstract

Cefaclor, a second-generation oral cephalosporin, is the most frequently prescribed cephalosporin in Korea. Studies, however, have yet to analyze the incidence of cefaclor-associated adverse drug reactions (ADRs), including hypersensitivity (HS), according to total national usage rates. This study aimed to investigate the incidence rates and clinical features of cefaclor ADRs reported to the Korean Adverse Event Reporting System (KAERS) and Health Insurance Review and Assessment Service (HIRA) database for the most recent 5 years. Reviewing the HIRA database, which contains information on all insurance claims, including prescribed medications and patient demographics, we identified the total number of individuals who had been prescribed cefaclor and other cephalosporins including 2^nd^ generation without cefaclor and 3^rd^ generation antibiotics from January 2014 to December 2018. Additionally, we retrospectively analyzed all ADRs reported to the KAERS for these drugs over the same study period. Incidence rates for ADRs, HS, and anaphylaxis to cefaclor were 1.92/10,000 persons, 1.17/10,000 persons, and 0.38/10,000 persons, respectively, lower than those to other 2^nd^ and 3^rd^ cephalosporins. Among all ADRs, HS (60.9% vs. 43.6% vs. 44.8%, *P* <0.001) and anaphylaxis (19.8% vs. 4.6% vs. 4.7%, *P* <0.001) were more common for cefaclor than for other 2^nd^ and 3^rd^ cephalosporins. Females, individuals under 65 years of age, concomitant use of drugs, and serious ADRs were more strongly associated with HS to cefaclor than with HS to other 2^nd^ and 3^rd^ cephalosporins. In a nationwide database for the Korean population, the incidence of cefaclor-induced ADRs, particularly HS and anaphylaxis, was high. Female sex, age younger than 65 years, and concomitant use of drugs may be associated with HS to cefaclor.

## Introduction

Adverse drug reactions (ADRs) are common and responsible for significant morbidity and mortality. The incidence of ADRs for all hospital admissions has been estimated at approximately 3–10%, varying greatly among reports, and ADRs pose a considerable public health problem [1–3]. ADRs were subdivided into type A and type B reactions as previously described, and type B reactions mainly included hypersensitivity reactions that were subdivided into immediate and delayed HS. Drug hypersensitivity (HS) is unpredictable, dose independent after the dose exceeding the threshold, and potentially life-threatening [4,5]. In addition, diagnosis of anaphylaxis was based on diagnostic criteria set forth in the 2011 World Allergy Organization Anaphylaxis Guidelines [6]. Several studies on the incidence of HS and/or anaphylaxis to cephalosporins have been conducted on large subjects [7,8], and past history of HS to penicillin or cephalosporin is the most important risk factor for reacting to cephalosporins [9]. However, data on the incidence thereof and results on risk factors for drug HS are unclear, because its diagnosis depends on a patient’s history and clinical manifestation. Additionally, studies have shown that the incidence of drug HS can be affected by various factors, such as study population, definition, and methods of data analysis [3,10].

Cefaclor is a second-generation oral cephalosporin and is reportedly the most frequently prescribed antibiotic among cephalosporins in Korea [11]. The prescription of cefaclor in Korea has continued to increase since 2015 according to Health Insurance Review and Assessment (HIRA) Service data [12], and cefaclor has been reported to be the most common causative drug of anaphylaxis in a tertiary care hospital in Korea [13]. Although studies on cefaclor-induced ADRs, HS, and/or anaphylaxis have been reported [14–18], the incidences of individual cefaclor associated-ADRs based on total national usage rates have not been analyzed. Accordingly, we sought to investigate the incidence rates and clinical features of cefaclor-induced ADRs in a nationwide database provided by the Korea Adverse Event Reporting System (KAERS) and HIRA for the most recent 5 years.

## Materials and methods

### Data source on drug prescriptions

We reviewed information from the HIRA database, which contains information on all medical insurance claims, including prescribed medications, for approximately 97% of the entire Korean population covered under the national health insurance system. The HIRA service provided data retrieved from medical facilities and pharmacies, with information on patient demographics, diagnoses, prescriptions, and procedures after de-identification. Therewith, we assessed the total number of individuals who had been prescribed cefaclor and other 2^nd^ (without cefaclor) and 3^rd^ cephalosporin antibiotics from January 2014 to December 2018.

### Data source on adverse drug reactions

Introduced in 1988, the KAERS is a spontaneous ADR reporting system in Korea and has been regulated by the Korea Institute of Drug Safety & Risk Management (KIDS) since 2012. ADR reports are submitted to KAERS primarily by regional pharmacovigilance centers, including general hospitals, and associations of pharmacists, pharmaceutical companies, health providers, and even patients via paper forms, telephone, fax, or electronically on the KIDS website using a standardized form. Culprit drugs are coded according to the Anatomical Therapeutic Chemical (ATC) classification system, and adverse events are coded according to World Health Organization Adverse Reaction Terminology (WHO-ART) which has been used in Korea since 2006.

### Classification and assessment of adverse drug reaction data

In this study, we retrospectively reviewed all ADRs to cefaclor and other 2^nd^ and 3^rd^ cephalosporins reported to the KAERS from January 2014 to December 2018. We identified HS and anaphylaxis using WHO-ART terms. Cases of HS were defined according to WHO-ART preferred terms (PTs) as follows: A) ADR reports wherein more than one code listed in S1 Table was present or B) ADR reports wherein two or more of the codes listed in S2 Table were present. Cases of anaphylaxis were defined according to WHO-ART PTs and System Organ Classes (SOCs) as follows (S3 Table): A) ADR reports coded “anaphylaxis” among SOCs; B) ADR reports coded “skin” and “cardiovascular” or “respiratory” among SOCs; and C) ADR reports coded with two or more SOCs of “skin”, “cardiovascular”, “respiratory”, and “gastrointestinal”.

All ADR cases included in the present study were assessed as being of possible, probable, or certain cause according to the WHO-Uppsala Monitoring Center criteria by healthcare professionals in regional pharmacovigilance centers. A serious adverse reaction was defined as “one requiring or prolonging hospitalization, causing congenital anomaly, resulting in persistent or significant disability, life-threatening, or resulting in death” [4]. We reviewed information on age, sex, type of reporters, concomitant medications, and indications of drug administration in each ADR case from the KAERS database. Suspected and concomitant medications were identified using ATC classification, and indications of drug administration were identified using International Classification of Diseases 10^th^ Revision (ICD-10). This study was approved by the institutional review boards of both Ajou University Hospital and Dong-A University Hospital (AJRIB-MED-MDB-19-231 and DAUHIRB-19-136), respectively, and all data were fully anonymized before we accessed them.

### Statistical analysis

Incidence rates for ADR, HS, and anaphylaxis to cefaclor, other 2^nd^ generation cephalosporins and 3^rd^ generation cephalosporins were calculated as for each category of ADRs in the KAERS database divided by the total number of individuals who had been prescribed these drugs in the HIRA database during the study period, and are expressed as incidence per 10,000 persons. Categorical variables are described as frequencies and proportions, and continuous variables are presented as means ± standard deviations and absolute numbers. Statistical significance was assessed using Student’s t-test for continuous variables and Pearson’s chi-squared test for categorical variables. *P*<0.05 was considered statistically significant. Statistical analyses were conducted using R program, version 3.6.3 (http://www.r-project.org).

## Results

The total numbers of prescriptions of cefaclor, other 2nd cephalosporins, and 3^rd^ cephalosporins during the study period were 35,902,648, 15,619,584, and 17,146,861 respectively, according to the HIRA database. Annual numbers of prescriptions of cefaclor and other 2^nd^ and 3^rd^ cephalosporins are shown in Table 1. Patients who had been prescribed cefaclor or other 2^nd^ and 3rd cephalosporins showed similar distribution of sex and age (Table 1). In total, 6,883, 12,800, and 22,294 cases of ADRs to cefaclor, other 2^nd^ cephalosporins, and 3rd cephalosporins, respectively, were reported in the KAERS database during the study period. All annual reports of ADRs to cefaclor, other 2^nd^ cephalosporins, and 3^rd^ cephalosporins increased continuously, and most of the ADRs to cefaclor, other 2^nd^ cephalosporins, and 3^rd^ cephalosporins were spontaneously reported by regional pharmacovigilance centers (cefaclor vs. other 2^nd^ cephalosporins; 98.3% vs. 96.7%, *P*<0.001, cefaclor vs. 3^rd^ cephalosporins; 98.3% vs. 95.5, *P*^^^<0.001) (Table 2). The proportion of females experiencing ADRs was statistically significantly higher for cefaclor than for other 2^nd^ and 3rd cephalosporins (cefaclor vs. other 2^nd^ cephalosporins; 65.5% vs. 58.5%, *P*<0.001, cefaclor vs. 3^rd^ cephalosporins; 65.5% vs. 52.1%, *P*^^^<0.001). Age was significantly younger for cefaclor ADRs than for 3rd cephalosporins ADRs (47.6±17.4 years vs. 49.3±23.4 years, *P*^^^<0.001), but no significant difference in age between cefaclor ADRs and other 2^nd^ cephalosporins (47.6±17.4 years vs. 47.2±19.2 years, *P* = 0.251). Cefaclor ADRs were more common in individuals aged 30 to 50 years than other 2^nd^ and 3^rd^ cephalosporins ADRs with statistical significance (30s: 15.3% vs. 14.1% vs. 9.8%, 40s: 19.3% vs. 15.8% vs. 11.0%, 50s: 23.0% vs. 19.4% vs. 16.3%) (Table 2).

**Table 1:** Prescriptions of cefaclor and other 2^nd^ and 3^rd^ cephalosporins in the Health Insurance Review and Assessment Service database in Korea for the most recent 5 years.

	Cefaclor N = 35,902,648 (%)	Other 2^nd^ cephalosporins N = 15,619,584 (%)	3^rd^ cephalosporins N = 17,146,861 (%)
**Female**	18,884,652 (52.6)	8,491,507 (54.4)	9,241,463 (53.9)
**Age, years**			
0–9	3,937,238 (11.0)	1,910,540 (12.2)	3,826,513 (22.3)
10–19	4,461,479 (12.4)	1,844,820 (11.8)	1,677,280 (9.8)
20–29	5,357,101 (14.9)	1,973,290 (12.6)	1,670,411 (9.7)
30–39	6,137,385 (17.1)	2,560,838 (16.4)	2,287,212 (13.3)
40–49	6,567,638 (18.3)	2,505,652 (16.0)	2,192,517 (12.8)
50–59	6,551,235 (18.2)	2,460,543 (15.8)	2,314,416 (13.5)
60–69	4,319,144 (12.0)	1,778,043 (11.4)	1,859,000 (10.8)
70–79	2,647,211 (7.4)	1,167,898 (7.5)	1,482,109 (8.6)
≥80	1,016,467 (2.8)	467,485 (3.0)	864,976 (5.0)
**Report year**			
2014	14,511,415	4,429,987	5,061,842
2015	14,346,403	4,483,135	5,223,740
2016	14,772,831	4,737,316	5,619,054
2017	14,572,493	4,714,094	5,506,605
2018	14,804,131	4,921,172	5,960,831

**Table 2:** Characteristics of adverse drug reaction cases to cefaclor and other 2^nd^ and 3^rd^ cephalosporins in the Korean Adverse Event Reporting System database for the most recent 5 years.

	Cefaclor N = 6,883 (%)	Other 2^nd^ cephalosporins N = 12,800 (%)	3^rd^ cephalosporins N = 22,294 (%)	*P-*value	*P*^*^-*^value
**Female sex**	4,508 (65.5)	7,482 (58.5)	11,618 (52.1)	<0.001	<0.001
Age*, years	47.6±17.4	47.2±19.2	49.3±23.4	0.251	<0.001
0–9	184 (2.7)	341 (2.7)	1,881 (8.4)	>0.999	<0.001
10–19	268 (3.9)	727 (5.7)	935 (4.2)	<0.001	0.289
20–29	554 (8.0)	1,419 (11.1)	1,732 (7.8)	<0.001	0.466
30–39	1,056 (15.3)	1,806 (14.1)	2,177 (9.8)	0.020	<0.001
40–49	1,331 (19.3)	2,017 (15.8)	2,452 (11.0)	<0.001	<0.001
50–59	1,586 (23.0)	2,480 (19.4)	3,736 (16.8)	<0.001	<0.001
60–69	1,086 (15.8)	1,943 (15.2)	3,629 (16.3)	0.276	0.334
70–79	477 (6.9)	1,217 (9.5)	3,135 (14.1)	<0.001	<0.001
≥80	129 (1.9)	372 (2.9)	1,502 (6.7)	<0.001	<0.001
Unknown	212 (3.1)	478 (3.7)	1,115 (5.0)	0.019	<0.001
**Report year**					
2014	755 (11.0)	2,048 (16.0)	3,367 (15.1)	<0.001	<0.001
2015	1,054 (15.3)	2,319 (18.1)	4,027 (18.1)	<0.001	<0.001
2016	1,333 (19.4)	2,782 (21.7)	4,457 (20.0)	<0.001	0.263
2017	1,696 (24.6)	2,954 (23.1)	4,889 (21.9)	0.015	<0.001
2018	2,045 (29.7)	2,697 (21.1)	5,554 (24.9)	<0.001	<0.001
**Reporter**					
Regional pharmacovigilance center	6,768 (98.3)	12,382 (96.7)	21,301 (95.5)	<0.001	<0.001
Manufacturer	26 (0.4)	7 (0.1)	160 (0.7)	<0.001	0.003
General hospital	89 (1.3)	407 (3.2)	832 (3.7)	<0.001	<0.001
Pharmacy	0 (0.0)	1 (0.0)	1 (0.0)	>0.999	>0.999
Others	0 (0.0)	3 (0.0)	0 (0.0)	0.506	N/A

* Data are presented as means ± standard deviations.

N/A; not available.

*P* value: Cefaclor vs. 2nd cephalosporins without cefaclor.

*P*^*^*^ value: Cefaclor vs. 3rd cephalosporins.

### Incidence rates of adverse drug reactions, hypersensitivity, and anaphylaxis to cefaclor and other 2^nd^ and 3^rd^ cephalosporins

**Fig 1:**
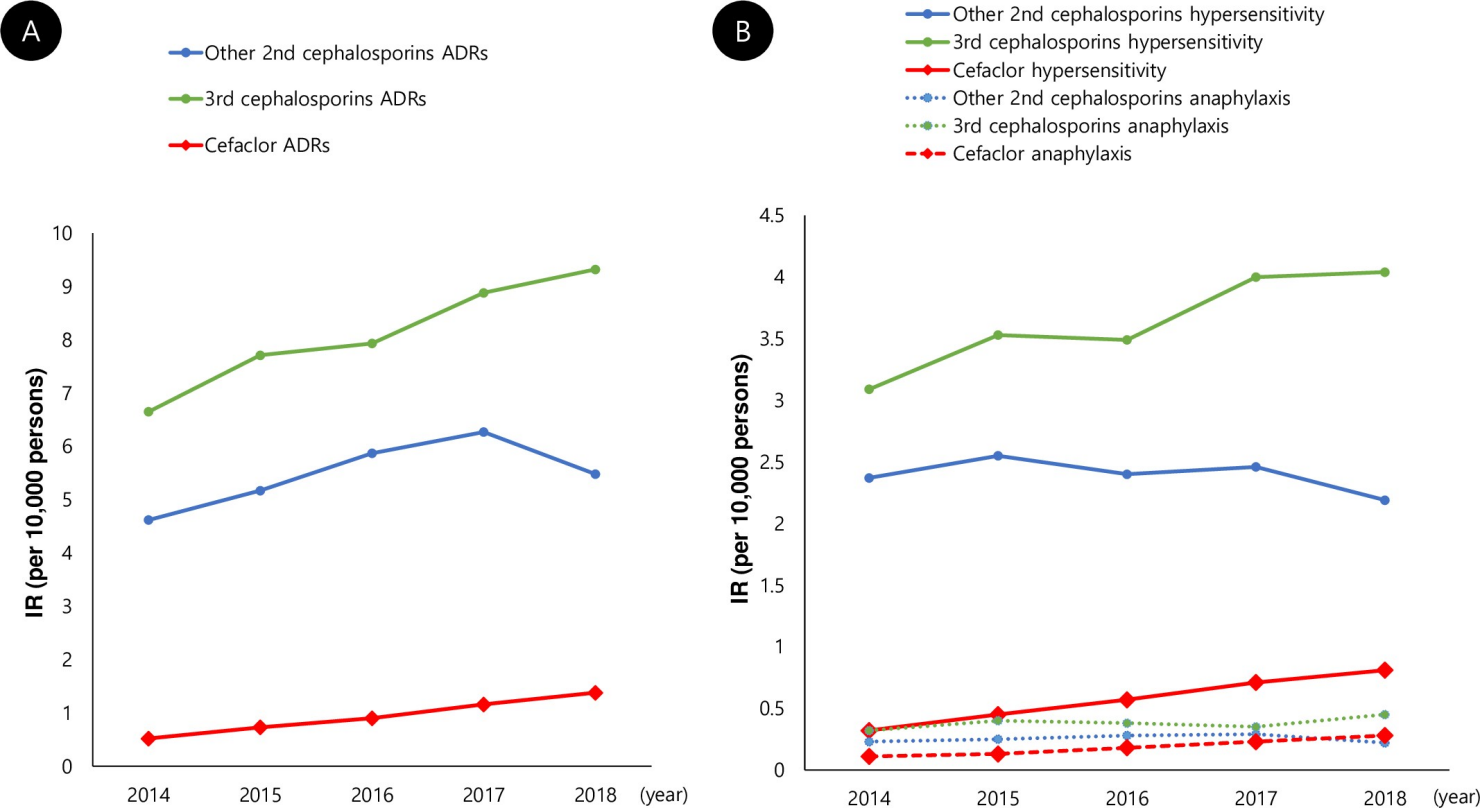
Annual incidence rates of adverse drug reactions, hypersensitivity, and anaphylaxis to cefaclor and other 2^nd^ and 3^rd^ cephalosporins during the study period. ADR: Adverse drug reaction.

**Fig 2:**
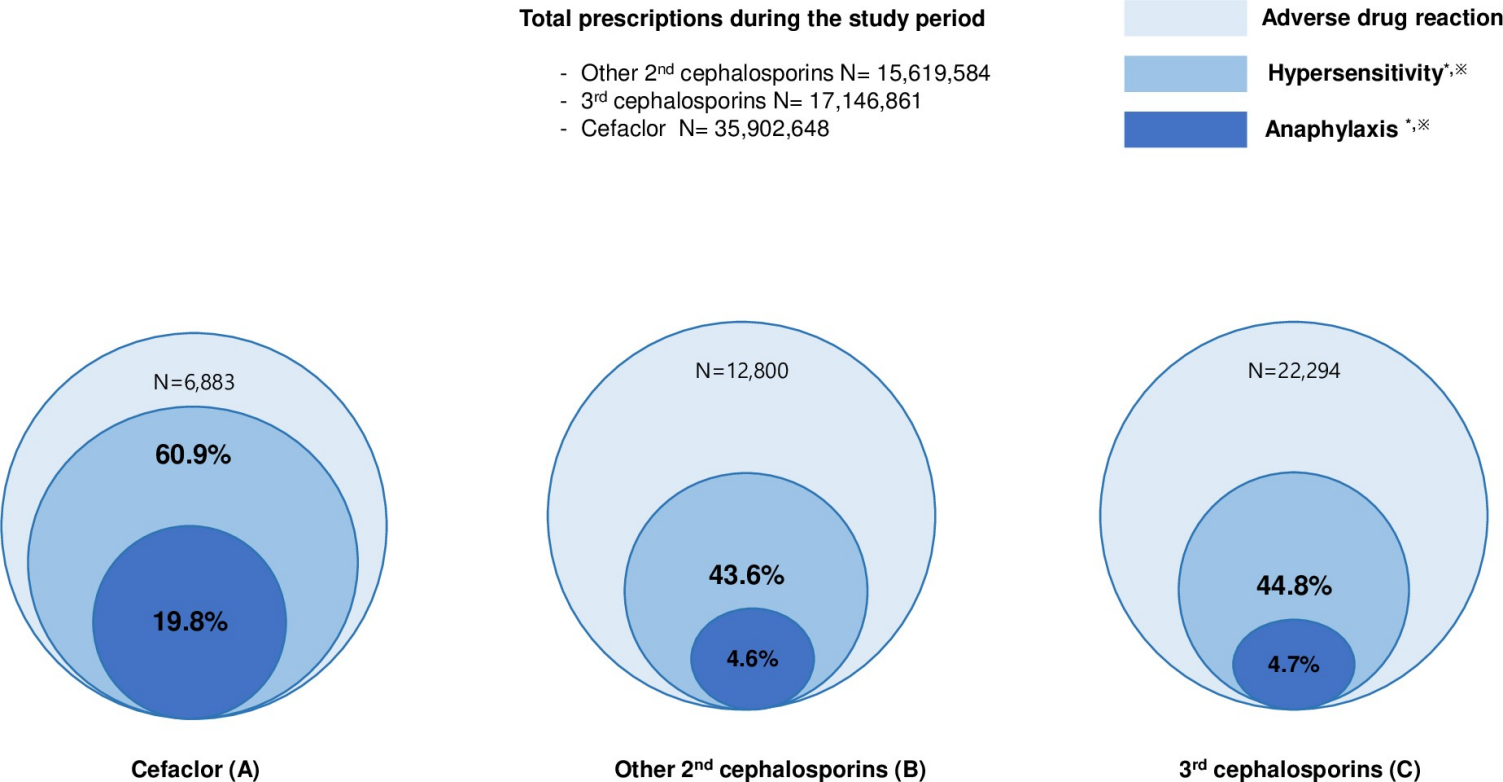
**Proportions of hypersensitivity and anaphylaxis among adverse drug reactions to cefaclor (A), other 2**^**nd**^ **cephalosporins (B), and 3**^**rd**^ **cephalosporins (C).** * *P*<0.001 (other 2^nd^ cephalosporins vs. cefaclor). ^※^ *P*^*^*^<0.001 (3^rd^ cephalosporins vs. cefaclor).

Incidence rates of ADRs to cefaclor, other 2^nd^ cephalosporins, and 3^rd^ cephalosporins during the study period were 1.92/10,000 persons, 8.19/10,000 persons, and 13.0/10,000 persons, respectively. Incidence rates for HS (and anaphylaxis) to cefaclor, other 2^nd^ cephalosporins, and 3^rd^ cephalosporins were 1.17/10,000 persons (0.38/10,000 persons), 3.57/10,000 persons (0.38/10,000 persons), and 5.82/10,000 persons (0.61/10,000 persons), respectively (Table 3). The majority of incidence rates for ADRs, HS, and anaphylaxis to cefaclor and 3^rd^ cephalosporins showed a tendency to increase gradually during the study period (Fig 1). The proportions of clinical types of ADRs were significantly different between cefaclor and other 2^nd^ and 3^rd^ cephalosporins (Fig 2): HS (60.9% vs. 43.6% vs. 44.8%) and anaphylaxis (19.8% vs. 4.6% vs. 4.7%) were more common for cefaclor than for other 2^nd^ and 3^rd^ cephalosporins with statistical significance.

**Table 3:** Incidence rates of adverse drug reactions, hypersensitivity and anaphylaxis to cefaclor and other 2^nd^ and 3^rd^ cephalosporins.

	Cefaclor	Other 2^nd^ cephalosporins	3^rd^ cephalosporins	*P-*value	*P*^*^*^-value
**ADR**	**IR, per 10,000 persons**				
Total	1.92	8.19	13.00	<0.001	<0.001
2014	0.52	4.62	6.65	<0.001	<0.001
2015	0.73	5.17	7.71	<0.001	<0.001
2016	0.9	5.87	7.93	<0.001	<0.001
2017	1.16	6.27	8.88	<0.001	<0.001
2018	1.38	5.48	9.32	<0.001	<0.001
**Hypersensitivity**	**IR, per 10,000 persons**				
Total	1.17	3.57	5.82	<0.001	<0.001
2014	0.32	2.37	3.09	<0.001	<0.001
2015	0.45	2.55	3.53	<0.001	<0.001
2016	0.57	2.40	3.49	<0.001	<0.001
2017	0.71	2.46	4.00	<0.001	<0.001
2018	0.81	2.19	4.04	<0.001	<0.001
**Anaphylaxis**	**IR, per 10,000 persons**				
Total	0.38	0.38	0.61	0.937	<0.001
2014	0.11	0.23	0.32	<0.001	<0.001
2015	0.13	0.25	0.40	<0.001	<0.001
2016	0.18	0.28	0.38	<0.001	<0.001
2017	0.23	0.29	0.35	0.020	<0.001
2018	0.28	0.22	0.45	0.012	<0.001

ADR: Adverse drug reaction, IR: Incidence rate.

*P* value: Cefaclor vs. 2nd cephalosporins without cefaclor.

*P*^*^*^ value: Cefaclor vs. 3rd cephalosporins.

### Comparison of clinical characteristics between hypersensitivity/anaphylaxis to cefaclor and other 2^nd^ and 3^rd^ cephalosporins

Females more commonly experienced HS to cefaclor than to other 2^nd^ and 3^rd^ cephalosporins. Mean age of 3^rd^ cephalosporins HS group was the oldest, followed by cefaclor HS, other 2^nd^ cephalosporins HS group, and so on. However, the proportion of individuals aged ≥65 years was statistically significantly lower for cases of cefaclor HS than for cases of other 2^nd^ cephalosporins and 3^rd^ cephalosporins HS (14.4% vs. 16.1% vs. 26.4%) (Table 4).

**Table 4:** Assessment and clinical characteristics of hypersensitivity to cefaclor and other 2^nd^ and 3^rd^ cephalosporins.

	Cefaclor HS N = 4,185 (%)	Other 2^nd^ cephalosporins HS N = 5,569 (%)	3^rd^ cephalosporins HS N = 9,988 (%)	*P* value	*P*^*^*^ value
**Females, n**	2,664 (63.7)	3,259 (59.2)	5,235 (52.4)	<0.001	<0.001
Age*, years	47.7±16.9	46.01±18.50	49.23±22.04	<0.001	<0.001
0–9	91 (2.2)	132 (2.4)	674 (6.7)	0.567	<0.001
10–19	164 (3.9)	340 (6.1)	443 (4.4)	<0.001	0.180
20–29	324 (7.7)	647 (11.6)	826 (8.3)	<0.001	0.309
30–39	648 (15.5)	893 (16.0)	1,086 (10.9)	0.477	<0.001
40–49	823 (19.7)	951 (17.1)	1,279 (12.8)	0.001	<0.001
50–59	989 (23.6)	1,076 (19.3)	1,780 (17.8)	<0.001	<0.001
60–69	672 (16.1)	811 (14.6)	1,681 (16.8)	0.045	0.270
70–79	263 (6.3)	432 (7.8)	1,332 (13.3)	0.006	<0.001
≥80	73 (1.7)	136 (2.4)	554 (5.5)	0.022	<0.001
Unknown	138 (3.3)	151 (2.7)	333 (3.3)	0.103	0.953
**Old age**					
≥ 65	604 (14.4)	896 (16.1)	2,641 (26.4)	0.027	<0.001
**Serious ADR**	852 (20.4)	287 (5.2)	591 (5.9)	<0.001	<0.001
Disability	4 (0.1)	2 (0.0)	0 (0.0)	0.445	0.011
Congenital anomaly	0 (0.0)	0 (0.0)	0 (0.0)	N/A	N/A
Life-threatening	118 (2.8)	53 (1.0)	79 (0.8)	<0.001	<0.001
Death	4 (0.1)	3 (0.1)	7 (0.1)	0.704	0.868
Need for or prolongation of hospitalization	180 (4.3)	84 (1.5)	238 (2.4)	<0.001	<0.001
Others	587 (14.0)	162 (2.9)	298 (3.0)	<0.001	<0.001
**Clinical manifestations**					
Anaphylaxis	1,370 (32.7)	593 (10.6)	1,041 (10.4)	<0.001	<0.001
Urticaria	1,130 (27.0)	1,753 (31.5)	3,293 (33.0)	<0.001	<0.001
Angioedema	322 (7.7)	116 (2.1)	214 (2.1)	<0.001	<0.001
**Most common indications of prescription**					
1st	Diseases of the respiratory system (J00-J99) 523 (12.5)	Neoplasms (C00-D48) 670 (12.0)	Certain infectious and parasitic diseases (A00-B99) 1,277 (12.3)		
2nd	Injury, poisoning and certain other consequences of external causes (S00-T88) 361 (8.6)	Injury, poisoning and certain other consequences of external causes (S00-T88) 460 (8.2)	Diseases of the respiratory system (J00-J99) 1,109 (11.1)		
3rd	Certain infectious and parasitic diseases (A00-B99) 239 (5.7)	Certain infectious and parasitic diseases (A00-B99) 427 (7.7)	Diseases of the digestive system (K00-K93) 959 (9.6)		
Unknown	2,039 (48.7)	1,171 (21.0)	1,667 (16.7)		
**Common concomitant medications**					
Analgesic drugs	455 (7.7)	177 (6.3)	240 (5.2)	0.021	<0.001
Antihistamine	511 (8.6)	178 (6.3)	138 (3.0)	<0.001	<0.001
Gastrointestinal agents	1,778 (30.0)	632 (22.4)	1,344 (29.3)	<0.001	0.409
NSAIDs	1,441 (24.3)	377 (13.4)	298 (6.5)	<0.001	<0.001
Respiratory agents	761 (12.9)	286 (10.2)	413 (9.0)	<0.001	<0.001

HS: Hypersensitivity; NSAIDs: Non-steroidal anti-inflammatory drugs; N/A: Not available.

* Data are presented as means ± standard deviations.

*P* value: Cefaclor vs. 2nd cephalosporins without cefaclor.

*P*^*^*^ value: Cefaclor vs. 3rd cephalosporins.

The indication for drug administration were not identified in 48.7% of cases of cefaclor HS, 21.0% of cases of other 2^nd^ cephalosporins HS, and 16.7% of cases of 3^rd^ cephalosporins HS. The three most common indications for cefaclor were diseases of the respiratory system (J00-J99, 12.5%), injury, poisoning and certain other consequences of external causes (S00-T88, 8.6%), and certain infectious and parasitic diseases (A00-B99, 5.7%). Those for other 2^nd^ cephalosporins use were neoplasms (ICD-code: C00-D49) (12.0%); injury, poisoning and certain other consequences of external causes (S00-T88, 8.2%); and certain infectious parasitic diseases (A00-B99, 7.7%). Those for 3^rd^ cephalosporins were certain infectious and parasitic diseases (A00-B99, 12.3%); diseases of the respiratory system (J00-J99, 11.1%); and diseases of the digestive system (K00-K93, 9.6%) (Table 4).

The five most common concomitant medications, including analgesic drugs (7.7% vs. 6.3% vs. 5.2%), antihistamine (8.6% vs. 6.3% vs. 3.0%), gastrointestinal agents (30.0% vs. 22.4% vs. 29.3%), nonsteroidal anti-inflammatory drugs (NSAIDs) (24.3% vs. 13.4% vs. 6.5%), and respiratory agents (12.9% vs. 10.2% vs. 9.0%) were significantly more often prescribed in cefaclor HS than in other 2^nd^ and 3^rd^ cephalosporins HS (Table 4).

Similar differences in mean age and age distribution were noted between cefaclor anaphylaxis and other 2^nd^ and 3^rd^ cephalosporins anaphylaxis. Females were more commonly observed in cefaclor anaphylaxis than in other 2^nd^ and 3^rd^ cephalosporins anaphylaxis (60.7% vs. 60.0%, 55.6%). All the concomitant medications were more often prescribed in cases of cefaclor anaphylaxis than in cases of other 2^nd^ and 3^rd^ cephalosporins anaphylaxis (Table 5).

**Table 5:** Assessment and clinical characteristics of anaphylaxis to cefaclor and other 2^nd^ and 3^rd^ cephalosporins.

	Cefaclor anaphylaxis N = 1,370 (%)	Other 2^nd^ cephalosporins anaphylaxis N = 593 (%)	3^rd^ cephalosporins anaphylaxis N = 1,041 (%)	*P* value	*P*^*^*^ value
**Females, n**	837 (60.7)	356 (60.0)	579 (55.6)	0.811	0.013
Age*, years	47.2±14.8	44.4±18.62	47.84±22.33	0.001	0.467
0–9	13 (0.9)	14 (2.4)	69 (6.6)	0.024	<0.001
10–19	45 (3.3)	46 (7.8)	71 (6.8)	<0.001	<0.001
20–29	94 (6.9)	73 (12.3)	88 (8.5)	<0.001	0.165
30–39	234 (17.1)	92 (15.5)	111 (10.7)	0.430	<0.001
40–49	318 (23.2)	104 (17.5)	135 (13.0)	0.006	<0.001
50–59	356 (26.0)	95 (16.0)	160 (15.4)	<0.001	<0.001
60–69	195 (14.2)	84 (14.2)	174 (16.7)	>0.999	0.105
70–79	51 (3.7)	45 (7.6)	123 (11.8)	<0.001	<0.001
≥80	15 (1.1)	9 (1.5)	57 (5.5)	0.576	<0.001
Unknown	49 (3.6)	31 (5.2)	53 (5.1)	0.115	0.084
**Old age**					
≥ 65	147 (10.7))	88 (14.8)	269 (25.8)	0.012	<0.001
**Clinical manifestations**					
Urticaria	388 (28.3)	138 (23.3)	229 (22.0)	0.024	0.001
Angioedema	180 (13.1)	25 (4.2)	43 (4.1)	<0.001	<0.001
**Most common indications of prescription**					
1st	Diseases of the respiratory system (J00-J99) 286 (20.9)	Certain infectious and parasitic diseases (A00-B99) 51 (8.4)	Certain infectious and parasitic diseases (A00-B99) 124 (11.9)		
2nd	Injury, poisoning and certain other consequences of external causes (S00-T88) 221 (16.1)	Diseases of the respiratory system (J00-J99) 50 (8.4)	Diseases of the respiratory system (J00-J99) 111 (10.7)		
3rd	Certain infectious and parasitic diseases (A00-B99) 120 (8.8)	Injury, poisoning and certain other consequences of external causes (S00-T88) 48 (8.3)	Diseases of the genitourinary system (N00-N99) 91 (8.7)		
Unknown	337 (24.6)	140 (23.6)	207 (19.9)		
**Common concomitant medications**					
Analgesic drugs	85 (7.9)	14 (5.7)	24 (5.7)	0.301	0.165
Antihistamine	56 (5.2)	6 (2.4)	12 (2.8)	0.095	0.064
Gastrointestinal agents	311 (28.9)	53 (21.6)	103 (24.3)	0.027	0.085
NSAIDs	325 (30.2)	40 (16.3)	24 (5.7)	<0.001	<0.001
Respiratory agents	121 (11.2)	15 (6.1)	33 (7.8)	0.024	0.059

NSAIDs: Non-steroidal anti-inflammatory drugs.

* Data are presented as means ± standard deviations.

*P* value: Cefaclor vs. 2nd cephalosporins without cefaclor.

*P*^*^*^ value: Cefaclor vs. 3rd cephalosporins.

Anaphylaxis (32.7% vs. 10.6% vs. 10.4%), angioedema (7.7% vs. 2.1% vs. 2.1%) were more frequently reported in cases of cefaclor HS, and urticaria was less frequent in cases of cefaclor HS than in cases of other 2^nd^ and 3^rd^ cephalosporins HS (27.0% vs. 31.5% vs. 33.0%). However, among cases of anaphylaxis, urticaria and angioedema were more frequently reported in the cefaclor group than in the other 2^nd^ and 3^rd^ cephalosporins group (urticaria; 28.3% vs. 23.3% vs. 22.0, angioedema; 13.1% vs. 4.2% vs. 4.1%), and all of those differences were statistically significant (Table 5).

Serious ADRs were more common for cefaclor HS than for other 2^nd^ and 3^rd^ cephalosporins HS (20.4% vs. 5.2% vs. 5.9%), with statistical significance. There were four (0.1%), three (0.1%), and seven (0.1%) deaths in cefaclor HS, other 2^nd^ cephalosporins HS, and 3^rd^ cephalosporins HS, respectively (Table 4).

## Discussion

This is the first national survey to estimate incidence rates of cefaclor ADR, HS, and anaphylaxis. In the current study, the number of prescriptions of cefaclor was very high which are nearly same as that of other cephalosporins, and this is consistent with previous reports for Korea [19,20]. While the incidence rates of ADR, HS, and anaphylaxis to cefaclor were lower than those to other 2^nd^ and 3^rd^ cephalosporins, the proportions of HS and anaphylaxis to cefaclor among cefaclor ADRs were significantly higher than the proportions thereof to other 2^nd^ and 3^rd^ cephalosporins ADRs. Overall, the incidence rates of ADR, HS and anaphylaxis to both cefaclor and 3^rd^ cephalosporins increased consistently over the study period, although there is the possibility that this merely reflects increases in spontaneous reporting to the KAERS.

Several studies on the incidence of HS and/or anaphylaxis have been conducted. In a retrospective population-based analysis [7] in the United States, physician-documented cephalosporin-associated anaphylaxis occurred in five oral exposures (95% confidence interval, 1/1,428,571–1/96,154) among 622,456 patients exposed to 901,908 courses of treatment with oral cephalosporins and in eight parenteral exposures (95% confidence interval, 1/200,000–1/35,971) among 326,867 patients exposed to 487,630 courses of treatment with parenteral cephalosporins. In a large multicenter retrospective cohort study [8] in Korea, the incidence of intravenous cephalosporins-induced anaphylaxis was 6.8 per 100,000 exposures, and this is similar to our results on the incidence rate of anaphylaxis to cephalosporins (0.82 per 10,000 persons). Meanwhile, several studies on cefaclor-induced anaphylaxis have, however, reported inconsistent results for various populations [15,16,21–24], and incidence rates of cefaclor HS and anaphylaxis are unclear. The incidence rate of cefaclor HS was reported at 1.1% among 3,000 patients taking cefaclor in a previous study by Kammer et al. [25], and this is higher than the incidence rate for cefaclor anaphylaxis (0.0038%) in our study.

Various risk factors for drug HS have been reported previously and have largely been divided into drug-related factors and host-related factors. Drug-related risk factors include the chemical properties and molecular weights of a drug, dose, route of administration, treatment duration, and frequency of exposure. Host-related factors include age, sex, atopy, specific genetic polymorphism, and comorbidity [3,26,27]. When we compared clinical characteristics for cefaclor and other 2^nd^ and 3^rd^ cephalosporins HS groups, we noted female sex and age under 65 years to be potential risk factors for cefaclor HS. Also, we found that the proportions of cases in patients aged 40 to 50 years were much higher for HS to cefaclor than for HS to other 2^nd^ and 3^rd^ cephalosporins. These results are comparable to previously reported risk factors for drug allergy, especially IgE-mediated hypersensitivity reactions [17,28].

Cefaclor has a simple structure composed of an aminobenzyl (R1) side chain and Cl at R2, and researchers have indicated that IgE binding determinants on cefaclor encircle the whole molecule [29]. This structure may be related with the relatively high proportion of HS and anaphylaxis to cefaclor compared to other 2^nd^ and 3^rd^ cephalosporins among ADRs to them. In addition, in our study, serious ADRs were significantly more common among cases of HS to cefaclor than to other 2^nd^ and 3^rd^ cephalosporins. Although we are unable to state that serious ADRs are a cefaclor-specific problems, we do suggest that cefaclor HS could potentially pose a significant socioeconomic burden and public health problem and that further research is needed to determine whether these severe reactions are associated with structural features of cefaclor.

The use of concomitant medications, including analgesic drugs, antihistamine, gastrointestinal agents, NSAIDs, and respiratory agents, were more frequently prescribed in cases of cefaclor HS than in cases of other 2^nd^ and 3^rd^ cephalosporins HS. These medications were generally prescribed to patients with respiratory disease, in particular upper respiratory infections, and diseases of the respiratory system were the most common indications for cefaclor administration in this study. This difference could affect the difference in concomitant medications between cefaclor HS and other 2^nd^ and 3^rd^ cephalosporins HS.

There are several limitations to this study. First, this study was conducted retrospectively for data reported to a national spontaneously reporting system, and there were some ADRs that were not reported in this study. In addition, prescriptions of cefaclor and other 2^nd^ and 3^rd^ cephalosporins from the HIRA database were ascertained from de-identified data, and we were unable to confirm the exact number of patients exposed to each drug. Therefore, the incidence rates of ADRs may not be exact. However, we compared the incidence rates of cefaclor ADRs with those of other 2^nd^ and 3^rd^ cephalosporins ADRs, which were estimated using same method. Although the exact incidences of ADRs were not obtained in this study, our results can emphasize relatively higher proportions of HS and anaphylaxis to cefaclor than to other 2^nd^ and 3^rd^ cephalosporins among ADRs to them using nationwide data. Second, the KAERS database contained almost all spontaneous ADR reports from mainly 27 pharmacovigilance centers, other general hospitals, pharmaceutical companies, and pharmacies. Therefore, assessment of ADRs, including causality and seriousness could differ depending on the reporter. To offset this difference as much as possible, healthcare specialists and/or professional pharmacists at regional pharmacovigilance centers conduct a final review before inclusion in the database. However, in this study, the proportions of urticaria in the clinical manifestations of anaphylaxis to cefaclor and other 2^nd^ and 3^rd^ cephalosporins were significantly lower than what is commonly known [30]. This may be because of missing information in the ADR reports. In addition, information on concomitant medications and indications for drug administration were not exact. Systematic approaches to improve the quality of the reports of ADRs are necessary. Finally, we could not evaluate potential risk factors for ADRs, including prior exposure history or exposed intensity, because the individual patients were not identified.

Notwithstanding, this is the first study to estimate incidence rates of cefaclor ADR, HS, and anaphylaxis from a nationwide pharmacovigilance database of the Korean population. Compared to other 2^nd^ cephalosporins and 3^rd^ cephalosporins, cefaclor induced relatively high proportions of HS and anaphylaxis among ADRs in this study. Female sex and age under 65 years and concomitant administration of drugs were found to be potential risk factors for cefaclor HS. Physicians should be cautious about prescribing cefaclor to patients who may be fatal to anaphylaxis or are at a relatively high risk of developing hypersensitivity reaction. Further studies to obtain the exact incidence rates of cefaclor ADRs and to confirm risk factors thereof are needed.

## Supporting information

S1 Table WHO-ART codes for hypersensitivity. (DOCX)

S2 Table WHO-ART codes for hypersensitivity. (DOCX)

S3 Table WHO-ART codes for anaphylaxis. (DOCX)
