# Supplementary material for: Cefaclor-induced hypersensitivity: Differences in the incidence of anaphylaxis relative to other 2nd and 3rd generation cephalosporins
Source: PLoS One. 2021 Jul 22;16(7):e0254898. doi: 10.1371/journal.pone.0254898 (PMC8297852; doi:10.1371/journal.pone.0254898)
Supplement: S1 Table — (DOCX) [file pone.0254898.s001.docx]

**S1 Table. WHO-ART codes for hypersensitivity**

| WHO-ART | ARRN | SEQ |
| --- | --- | --- |
| Immediate type hypersensitivity reaction grade I | 2237 | 003 |
| Documented hypersensitivity to administered drug | 2268 | 001 |
| Rash acneiform | 0001 | 004 |
| Angioedema | 0003 | 001 |
| Quincke’s oedema | 0003 | 003 |
| Giant urticaria | 0003 | 004 |
| Angioneurotic oedema | 0003 | 005 |
| Giant hives | 0003 | 006 |
| Angioneurotic oedema aggravated | 0003 | 007 |
| Angioedema aggravated | 0003 | 008 |
| Epidermal necrolysis | 0013 | 001 |
| Dermatitis necrotising | 0013 | 003 |
| Lyell syndrome | 0013 | 004 |
| Toxic epidermal necrolysis | 0013 | 005 |
| Erythema multiforme | 0014 | 001 |
| Erythema annulare | 0014 | 003 |
| Erythema nodosum | 0015 | 001 |
| Itching | 0024 | 003 |
| Pruritus | 0024 | 001 |
| Rash | 0027 | 001 |
| Eruption | 0027 | 004 |
| Itching | 0027 | 003 |
| Toxicoderma | 0027 | 005 |
| Dermatitis medicamentosa | 0027 | 009 |
| Skin eruption | 0027 | 002 |
| Erythema | 0028 | 003 |
| Rash erythematous | 0028 | 001 |
| Rash maculo-papular | 0030 | 001 |
| Measly rash | 0030 | 003 |
| Morbilliform rash | 0030 | 004 |
| Macular rash | 0030 | 005 |
| Papular rash | 0030 | 006 |
| Papulosquamous rash | 0030 | 007 |
| Papulovesicular rash | 0030 | 008 |
| Vesicobullous rash | 0030 | 010 |
| Vesicopustular rash | 0030 | 011 |
| Vesicopustular rash aggravated | 0030 | 012 |
| Rash pustular | 0032 | 001 |
| Stevens Johnson Syndrome | 0042 | 001 |
| Erythema multiforme severe | 0042 | 003 |
| Mucocutaneous ulceration | 0042 | 004 |
| Urticaria | 0044 | 001 |
| Urticaroa vesiculosa | 0044 | 003 |
| Wheals | 0044 | 004 |
| Hives | 0044 | 005 |
| Urticaria aggravated | 0044 | 006 |
| Urticaria pigmentosa | 0044 | 008 |
| Urticaria localized | 0044 | 009 |
| Acute urticaria | 0045 | 001 |
| Systemic erythematous rash | 0081 | 011 |
| Vasculitis | 0085 | 001 |
| Vasculitis allergic | 0086 | 001 |
| Purpura allergic | 0460 | 001 |
| Purpura anaphylactoid | 0460 | 003 |
| Henoch-Schonlein purpura | 0460 | 004 |
| Purpura vascular allergic | 0460 | 005 |
| Allergic vascular purpura | 0460 | 006 |
| Anaphylactoid vascular purpura | 0460 | 007 |
| Allergic reaction | 0712 | 001 |
| Hypersensitivity | 0712 | 004 |
| Anaphylactic shock | 0713 | 001 |
| Immediate type hypersensitivity reaction grade IV | 0713 | 002 |
| Anaphylactoid reaction | 0714 | 001 |
| Fever | 0725 | 001 |
| Drug fever | 0725 | 006 |
| Bullous eruption | 0871 | 001 |
| Fixed eruption | 1249 | 001 |
| Rash vesicular | 1443 | 001 |
| Vesicular eruption | 1443 | 003 |
| Mucosa vesicle | 1443 | 004 |
| Varicellar-like eruption | 1443 | 005 |
| AGEP | 1846 | 003 |
| Anaphylaxis | 2237 | 002 |
| Anaphylactic reaction | 2237 | 001 |
| Immediate type hypersensitivity reaction grade II | 2237 | 004 |
| Immediate type hypersensitivity grade III | 2237 | 005 |
| Epidermolysis bullosa | 2259 | 001 |
| Drug hypersensitivity syndrome | 2309 | 001 |
| DRESS Syndrome | 2309 | 002 |

ARRN: adverse reaction record number; SEQ: sequence number, WHO-ART; World Health Organization- adverse reaction terminology
