# Supplementary material for: Cefaclor-induced hypersensitivity: Differences in the incidence of anaphylaxis relative to other 2nd and 3rd generation cephalosporins
Source: PLoS One. 2021 Jul 22;16(7):e0254898. doi: 10.1371/journal.pone.0254898 (PMC8297852; doi:10.1371/journal.pone.0254898)
Supplement: S2 Table — (DOCX) [file pone.0254898.s002.docx]

**S2 Table. WHO-ART codes for hypersensitivity**

| WHO-ART | ARRN | SEQ |
| --- | --- | --- |
| Neck oedema | 0003 | 009 |
| Dizziness | 0101 | 001 |
| Faintness | 0101 | 003 |
| Headache | 0109 | 001 |
| Smarting | 0137 | 013 |
| Tingling skin | 0137 | 015 |
| Electric shock sensation | 0137 | 021 |
| Consciousness decreased | 0151 | 009 |
| Altered state of consciousness | 0151 | 011 |
| Vertigo | 0158 | 001 |
| Drowsiness | 0197 | 008 |
| Flushing | 0207 | 001 |
| Bradycardia | 0208 | 001 |
| Blood pressure systolic decrease | 0210 | 011 |
| Hypotension | 0212 | 001 |
| Pallor | 0220 | 001 |
| Syncope | 0223 | 001 |
| Tachycardia | 0224 | 001 |
| Hyperaemia | 0225 | 003 |
| Conjunctival hyperaemia | 0238 | 002 |
| Conjunctival congestion | 0238 | 004 |
| Vision abnormal | 0257 | 001 |
| Vision decreased | 0257 | 003 |
| Heartburn | 0279 | 007 |
| GI distress | 0279 | 014 |
| Oral ulceration | 0328 | 005 |
| Tongue oedema | 0331 | 001 |
| Oedema | 0398 | 001 |
| Oedema generalised | 0400 | 001 |
| Shock | 0499 | 003 |
| Wheezes | 0511 | 006 |
| Coughing | 0513 | 001 |
| Dyspnoea | 0514 | 001 |
| Breath shortness | 0514 | 003 |
| Respiration labored | 0514 | 005 |
| Breath difficult | 0514 | 008 |
| Throat irritation | 0521 | 003 |
| Laryngeal oedema | 0522 | 005 |
| Laryngotracheal oedema | 0522 | 006 |
| Tachypnoea | 0536 | 003 |
| Oxygen saturation decreased | 0537 | 005 |
| Nose congestion | 0539 | 004 |
| Rhinorrhoea | 0539 | 006 |
| Sneezing excessive | 0539 | 007 |
| Nose oedema | 0539 | 008 |
| Nasal congestion | 0539 | 011 |
| Nasal dryness | 0539 | 012 |
| Stridor | 0542 | 001 |
| Eosinophilia | 0571 | 001 |
| Face oedema | 0602 | 001 |
| Chest pain | 0718 | 001 |
| Chest discomfort | 0718 | 003 |
| Thoracic pain | 0718 | 012 |
| Chest distress | 0718 | 013 |
| Pyrexia | 0725 | 004 |
| Shivering | 0731 | 003 |
| Oedema periorbital | 1009 | 001 |
| Oedema orbital | 1009 | 002 |
| Oedema eyelid | 1009 | 003 |
| Skin test positive | 1058 | 004 |
| Allergy test positive | 1058 | 005 |
| Skin peeling | 1199 | 003 |
| Oedema pharynx | 1395 | 001 |
| Oedema mouth | 1485 | 001 |
| Oedema circumoral | 1485 | 006 |
| Burning sensation | 1491 | 003 |
| Feeling of warmth | 1705 | 005 |
| Hot flushes | 1705 | 008 |
| Hyperpnoea | 1711 | 001 |
| Pharyngeal disorder | 2211 | 001 |
| Pruritus eyelid | 2253 | 003 |
