# Supplementary material for: Cefaclor-induced hypersensitivity: Differences in the incidence of anaphylaxis relative to other 2nd and 3rd generation cephalosporins
Source: PLoS One. 2021 Jul 22;16(7):e0254898. doi: 10.1371/journal.pone.0254898 (PMC8297852; doi:10.1371/journal.pone.0254898)
Supplement: S3 Table — (DOCX) [file pone.0254898.s003.docx]

**S3 Table. WHO-ART codes for anaphylaxis**

|  | SOC | ARRN |
| --- | --- | --- |
| Anaphylaxis | 1810 | 2237, 713-714, (712 or 1027 or 1058) & (212 or 213) |
| Skin | 0100 | 3, 7, 8, 24-28, 43-45, 1123, 1129, 207, 1009, 602 |
| Cardiovascular | 1010 | 108, 212-213, 220-221, 223-225, 229, 496, 499, 501-503, 1762, 1899, 398-401, 716, 718-719, 929 |
| Respiratory | 1100 | 144, 507, 511, 513-514, 519, 521-522, 536-537, 539, 541-542, 1367, 1466, 1490, 1565, 1711, 1749 |
| Gastrointestinal | 0600 | 268, 205, 228 |
